# Phosphorylation of Akt at Thr308 regulates p‐eNOS Ser1177 during physiological conditions

**DOI:** 10.1002/2211-5463.13194

**Published:** 2021-06-09

**Authors:** Xiao‐xue Liang, Rui‐yu Wang, Yong‐zheng Guo, Zhe Cheng, Ding‐yi Lv, Ming‐hao Luo, An He, Su‐xin Luo, Yong Xia

**Affiliations:** ^1^ Division of Cardiology The First Affiliated Hospital of Chongqing Medical University China; ^2^ Institute of Life Science Chongqing Medical University China

**Keywords:** Akt, endothelial nitric oxide synthase, nitric oxide, phosphorylation

## Abstract

Endothelial nitric oxide synthase (eNOS)‐derived nitric oxide (NO) plays a crucial role in maintaining vascular homeostasis. As a hallmark of eNOS activation, phosphorylation of eNOS at Ser1177 induced by activated protein kinase B (PKB/Akt) is pivotal for NO production. The complete activation of Akt requires its phosphorylation of both Thr308 and Ser473. However, which site plays the main role in regulating phosphorylation of eNOS Ser1177 is still controversial. The purpose of the present study is to explore the specific regulatory mechanism of phosphorylated Akt in eNOS activation. Inhibition of Akt Thr308 phosphorylation by a specific inhibitor or by siRNA *in vitro* led to a decrease in eNOS phosphorylation at Ser1177 and to lower NO concentration in the cell culture medium of HUVECs. However, inhibiting p‐Akt Ser473 had no effect on eNOS phosphorylation at Ser1177. Next, we administered mice with inhibitors to downregulate p‐Akt Ser473 or Thr308 activity. Along with the inhibition of p‐Akt Thr308, vascular p‐eNOS Ser1177 protein was simultaneously downregulated in parallel with a decrease in plasma NO concentration. Additionally, we cultured HUVECs at various temperature conditions (37, 22, and 4 °C). The results showed that p‐Akt Ser473 was gradually decreased in line with the reduction in temperature, accompanied by increased levels of p‐Akt Thr308 and p‐eNOS Ser1177. Taken together, our study indicates that the phosphorylation of Akt at Thr308, but not at Ser473, plays a more significant role in regulating p‐eNOS Ser1177 levels under physiological conditions.

## Graphical abstract

Endothelial nitric oxide synthase (eNOS) activation is regulated by Akt signaling; however, the specific regulatory mechanisms still remain unclear. We found that eNOS phosphorylation at Ser1177 and nitric oxide production were regulated by p‐Akt Thr308 but not by p‐Akt Ser473, both *in vitro* and *in vivo*. Therefore, we conclude that eNOS activation is mainly dependent on the phosphorylation of Akt at Thr308.

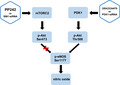

**Abbreviations**
AMPK: AMP‐dependent protein kinase
ANOVA: analysis of variance
CaM kinase II: calmodulin kinase II
DMSO: dimethyl sulfoxide
ECGS: endothelial cell growth supplement
eNOS: endothelial nitric oxide synthase
GRK2: G protein‐coupled receptor kinase 2
HUVECs: primary human umbilical vein endothelial cells
mTOR: mammalian target of rapamycin
mTORC2: mammalian target of rapamycin complex 2
NO: nitric oxide
PDK1: phosphoinositide‐dependent kinase‐1
PI3K: phosphatidylinositol‐3‐kinase
PIP3: phosphatidylinositol‐(3,4,5)‐trisphosphate
PKA: protein kinase A
PKB/Akt: protein kinase B
PKC: protein kinase C
PKG: protein kinase G
SE: standard error
siRNA: small interfering RNA

Nitric oxide (NO) is an essential endothelium‐derived relaxing factor which has versatile functions such as mediating vascular vasodilation, preventing vascular remodeling and blood coagulation, as well as maintaining the normal function of endothelial cells [1, 2, 3, 4]. The synthesis of NO is mainly catalyzed by endothelial nitric oxide synthase (eNOS) in vascular endothelial cells [5, 6]. eNOS activation is mainly regulated by multiple post‐translational modifications [7], including protein phosphorylation, acetylation, protein–protein interactions, and subcellular localization [8, 9, 10, 11]. Among them, the phosphorylation of eNOS plays the most important role in mediating eNOS activation. Ser1177 is a major positive regulatory site of eNOS, and its phosphorylation leads to the enhancement of eNOS activity and NO production, while Thr495 is a negative regulatory site which can diminish eNOS activation [12, 13, 14].

The phosphatidylinositol‐3‐kinase (PI3K)/protein kinase B (PKB/Akt)/eNOS signaling pathway is an important signal transduction axis that is involved in the synthesis of endogenous NO [7]. Activated PI3K facilitates the second messenger phosphatidylinositol‐(3,4,5)‐trisphosphate (PIP3) production, which can bind to Akt with the pleckstrin homology domain to induce conformational change and phosphorylation of Akt at Thr308 and Ser473 sites [15]. The phosphorylated Akt gains its enzymatic activity and then contributes to the activation of eNOS [16].

There are two different phosphorylated sites of Akt, namely Thr308 and Ser473. Phosphoinositide‐dependent kinase‐1 (PDK1) which locates on the cell membrane phosphorylates the site of Thr308 [17, 18], while mammalian target of rapamycin complex 2 (mTORC2) phosphorylates the site of Ser473 [19, 20]. However, which phosphorylated site of Akt is more effective for activating eNOS is still controversial. In fact, some studies only detect p‐Akt Ser473, while the other detects both of them [21, 22, 23, 24]. Thus, it is warranted to unify which one is the appropriate regulator of p‐eNOS ser1177.

In this study, we hypothesized that phosphorylation of Akt Thr308 is more crucial in regulating p‐eNOS Ser1177 than Ser473. To verify this hypothesis, we inhibited the phosphorylated levels of Akt at both sites of Thr308 and Ser473, and then, p‐eNOS Ser1177 expression was detected both *in vitro* and *in vivo*.

## Materials and methods

### Materials

Primary human umbilical vein endothelial cells (HUVECs) were from the institute of life science, Chongqing Medical University (Chongqing, China). Cell culture materials were purchased from Invitrogen (Carlsbad, CA, USA). Growth media were obtained from Gibco (Grand Island, NY, USA). GSK2334470 and PP242 were products of Sigma (St. Louis, MO, USA). Antibodies against p‐eNOS Ser1177, total‐Akt, p‐Akt Ser473, p‐Akt Thr308, PDK1, and SIN1 were purchased from Cell Signaling Technology (Beverly, MA, USA). Antibodies against total‐eNOS, p‐eNOS Thr495, and β‐actin were obtained from Millipore (Temecula, California).

### Cell culture and transfection

HUVECs were cultured in medium 199 (Invitrogen) with 20% fetal bovine serum (FBS; Invitrogen), 20 µg·mL^−1^ endothelial cell growth supplement (ECGS), 2 mm glutamine and 0.05 mg·mL^−1^ heparin, 1% penicillin and streptomycin. Passages at 5–8 were used for experiments. All cells were cultured in a humidified incubator at 37 °C with 5% CO_2_.

### Small interfering RNA (siRNA)

HUVECs were seeded in 6‐well plates. When the confluence reached 30%, siRNA oligonucleotides (100 nm) were transfected into cells by using Opti‐MEM (Invitrogen) and Lipofectamine 3000 (Invitrogen). siRNA targeting SIN1 and PDK1 were purchased from Biomic (Nanjing, China), and their target sequences were 5'‐GGUAUUAGAAGACGCUCAAdTdT‐3' and 5'‐CCUUCUUUGUUAAGCUUUAdTdT‐3', respectively. After 48 h of transfection, cells were harvested.

### Animals

Male C57BL/6J mice aged 6–8 weeks were purchased from the Experimental Animal Center of Chongqing Medical University (Chongqing, China). All animal studies were conducted with the ethical approved from the Institutional Ethics Committee of Chongqing Medical University [Animal permit No. SYXK (Chongqing) 2018‐0003] and the State Science and Technology Commission of China. Mice were housed in ventilated cages with a controlled environment (20–25 °C, 40–60% relative humidity, 12/12‐h light/dark cycles). Food and water were provided *ad libitum*.

### Animal studies

GSK2334470 (40 mg·kg^−1^) and PP242 (5 mg·kg^−1^) were administered to mice by intraperitoneal injection, respectively. In control group, mice were treated with equal volume of dimethyl sulfoxide (DMSO). After treatments for 6 h, mice were anesthetized to death by an overdose of pentobarbital sodium (150 mg·kg^−1^). The mesenteric artery was quickly removed from mice and washed by cold PBS.

### Western blotting

Cells and mesenteric artery of mice were lysed in the RIPA (Beyotime Biotechnology, JiangSu, China) containing 1% protein inhibitor and 1% phosphatase inhibitors (Gibco) on the cracker (4 °C) for 30 and 120 min, respectively, then centrifuged at 13 700 ***g*** for 15 min, and the supernatant was harvested. The protein concentration was measured by Bradford protein assay. Lysate supernatants were denatured by boiling in SDS sample buffer. The proteins were fractionated by SDS/PAGE on 8% gradient gels (Invitrogen) and then transferred to PVDF membranes (Bio‐Rad, Hercules, CA, USA). After blocking, the membranes were reacted with the appropriate primary antibodies at 4 °C overnight. After washing with TBST buffer solution, the membranes were incubated with secondary antibody (goat anti‐rabbit; Millipore) for 90 min at room temperature. Signals were developed on films employing the enhanced chemiluminescence technique (SuperSignal West Pico; Pierce) or visualized using ECL kit (Advansta, San Jose, CA, USA) by software with image lab software (Bio‐Rad).

### Determination of NO

The concentration of NO in the medium of cultured HUVECs was measured with the Total Nitric Oxide Assay Kit (Beyotime). Blood samples of mice were collected and centrifuged at 1200 ***g*** for 10 min. The supernatant plasma was harvested for NO detection using a commercially available NO assay ELISA kits (Sbjbio, Nanjing, China).

### Statistical analysis

All data were presented as means ± standard error (SE). Comparisons between two groups were conducted using a two‐tailed unpaired Student’s *t*‐test, and comparisons among multiple groups were made using one‐way analysis of variance (ANOVA) followed by Tukey's test. The statistical analysis was performed using graphpad prism software version 7.0 (San Diego, CA, USA). Differences were considered statistically significant at a value of *P* < 0.05.

## Results

### Inhibiting phosphorylation of Akt at Thr308 accompanied by decreased p‐eNOS Ser1177 expression

**Fig. 1:**
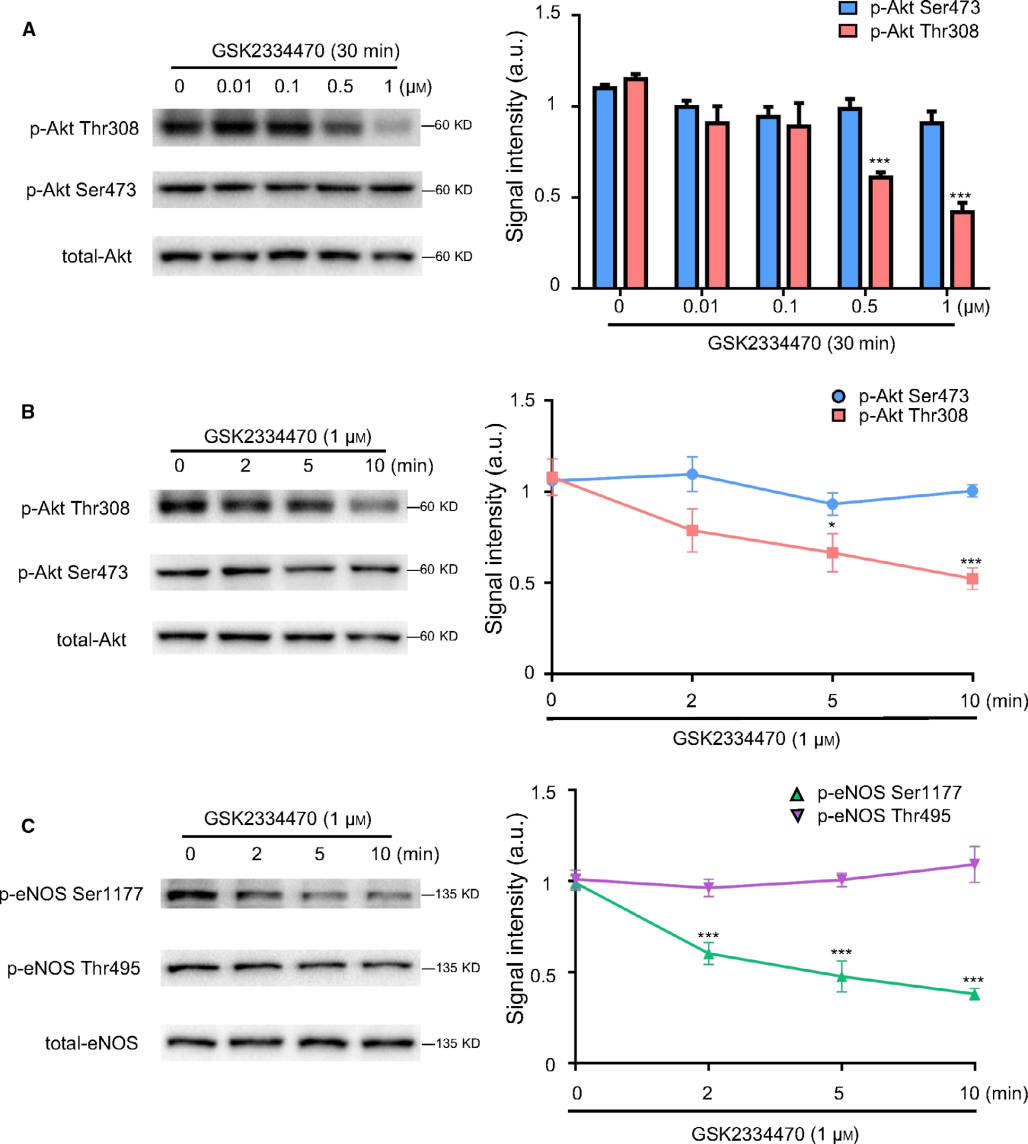
Inhibiting phosphorylation of Akt at Thr308 accompanied by decreased p‐eNOS Ser1177 expression. HUVECs were treated with GSK2334470 at different concentrations (0.01, 0.1, 0.5, 1 μm) or for different durations (2, 5, 10 min) to explore the effects of GSK2334470 on p‐eNOS Ser1177 expression. (A) Western blot and quantitative analysis of the protein expressions of p‐Akt Thr308, p‐Akt Ser473, and total‐Akt in HUVECs treated with different concentrations of GSK2334470 for 30 min. (B) Western blot and quantitative analysis of the protein expressions of p‐Akt Thr308, p‐Akt Ser473, and total‐Akt in HUVECs treated with GSK2334470 (1 μm) for different durations. (C) Western blot and quantitative analysis of the protein expressions of p‐eNOS Ser1177, p‐eNOS Thr495, and total‐eNOS in HUVECs treated with GSK2334470 (1 μm) for different durations. The relative levels of p‐Akt Thr308, p‐Akt Ser473, p‐eNOS Ser1177, and p‐eNOS Thr495 were quantified as the ratios of p‐Akt Thr308/Akt, p‐Akt Ser473/Akt, p‐eNOS Ser1177/eNOS, and p‐eNOS Thr495/eNOS, respectively. Data were expressed as mean ± SE, *n* = 4. Data were analyzed with one‐way ANOVA followed by Tukey's multiple comparisons test. *P < 0.05 vs. Control group; ***P < 0.001 vs. Control group.

PDK1 is an upstream molecule that phosphorylates Akt at Thr308 [25]. To explore the relationship between p‐Akt Thr308 and p‐eNOS Ser1177, GSK2334470, a selective inhibitor of PDK1, was used to suppress the phosphorylation of Akt Thr308. As shown in Fig. 1A, GSK2334470 (0–1 μm) dose‐dependently decreased p‐Akt Thr308, whereas the level of p‐Akt Ser473 was not significantly affected. Then, HUVECs were subjected to an appropriate concentration of GSK2334470 at 1 μm with a time gradient (0–10 min). As shown in Fig. 1B, the expression of p‐Akt Thr308 dropped sharply in a time‐dependent manner and reached to significant statistical difference after 5 min. However, the expression of p‐Akt Ser473 remained unchanged. Subsequently, we found that inhibiting p‐Akt Thr308 by GSK2334470 downregulated p‐eNOS Ser1177 expression to 50% at 5 min. In contrast, the expression of p‐eNOS Thr495 remained unchanged (Fig. 1C). Taken together, these data suggested that the phosphorylation of Akt Thr308 might potentially regulate p‐eNOS Ser 1177 expression.

### p‐eNOS Ser1177 was independent of phosphorylation of Akt at Ser473

**Fig. 2:**
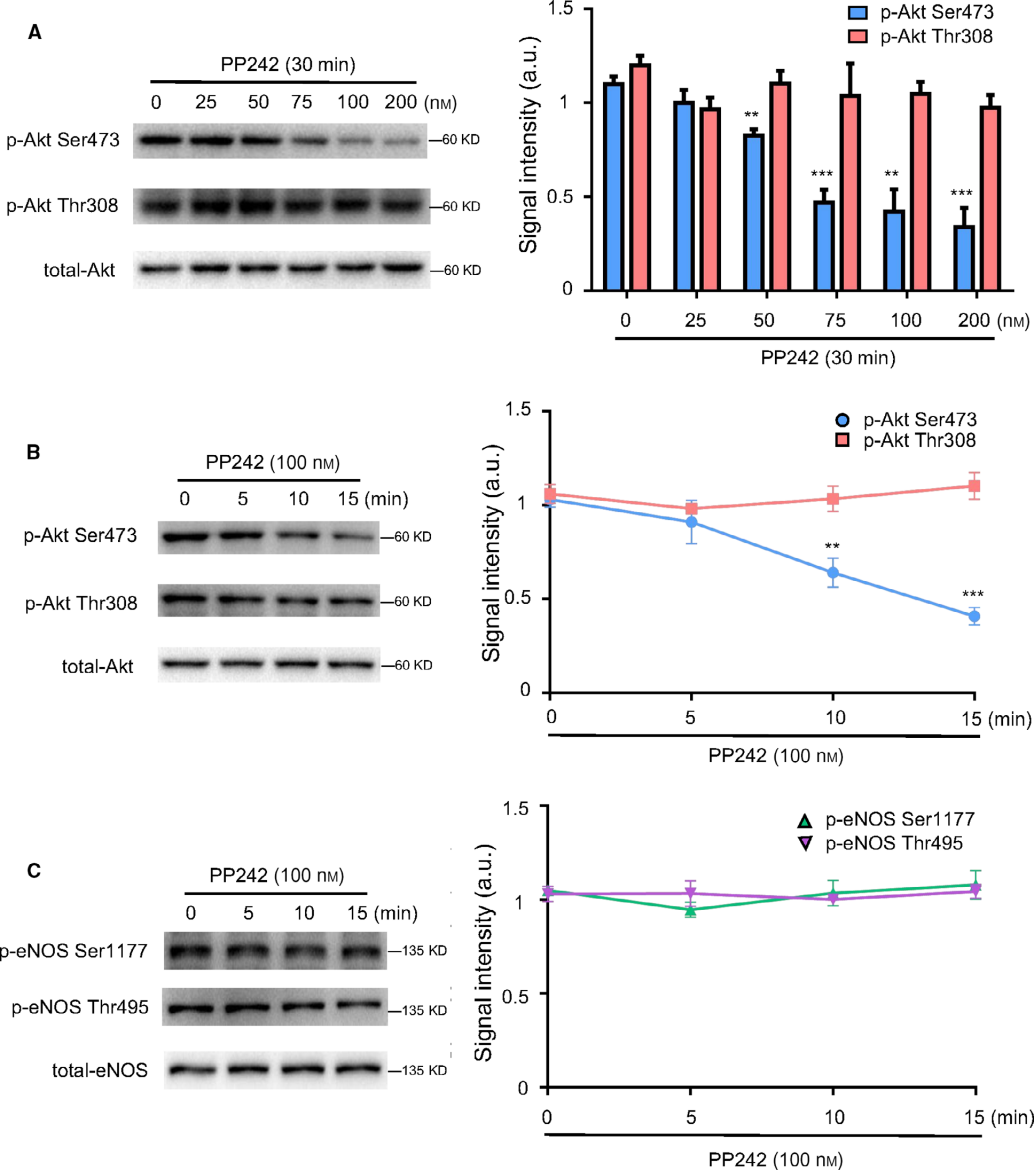
p‐eNOS Ser1177 was independent of phosphorylation of Akt at Ser473. HUVECs were treated with PP242 at different concentrations (25, 50, 75, 100, and 200 nm) or for different durations (5, 10, and 15 min) to explore the effects of p‐Akt Ser473 on p‐eNOS Ser1177 expression. (A) Western blot and quantitative analysis of the protein expressions of p‐Akt Thr308, p‐Akt Ser473, and total‐Akt in HUVECs treated with different concentrations of PP242 for 30 min. (B) Western blot and quantitative analysis of the protein expressions of p‐Akt Thr308, p‐Akt Ser473, and total‐Akt in HUVECs treated with PP242 (100 nm) for different durations. (C) Western blot and quantitative analysis of the protein expressions of p‐eNOS Ser1177, p‐eNOS Thr495, and total‐eNOS in HUVECs treated with GSK2334470 (100 nm) for different durations. The relative levels of p‐Akt Thr308, p‐Akt Ser473, p‐eNOS Ser1177, and p‐eNOS Thr495 were quantified as the ratios of p‐Akt Thr308/Akt, p‐Akt Ser473/Akt, p‐eNOS Ser1177/eNOS, and p‐eNOS Thr495/eNOS, respectively. Data were expressed as mean ± SE, *n* = 4. Data were analyzed with one‐way ANOVA followed by Tukey's multiple comparisons test. **P < 0.01 vs. Control group; ***P < 0.001 vs. Control group.

PP242 is a selective inhibitor of mTOR which regulates the phosphorylation of Akt Ser473 [26]. To further verify the role of p‐Akt Ser473 in p‐eNOS Ser1177, HUVECs were exposed to PP242 with increasing concentration gradient (0–200 nm). As shown in Fig. 2A, a dose‐dependently downregulation of p‐Akt Ser473 was observed in HUVECs upon PP242 treatments, but no significant changes were shown in the expressions of p‐Akt Thr308 and total‐Akt. Subsequently, HUVECs were treated with PP242 (100 nm) for different time. As expected, the expression of p‐Akt Ser473 tended to drop over time and significantly decreased after 10 min (Fig. 2B), while the expression of p‐Akt Thr308 remained unchanged. Then, we detected the levels of phosphorylated eNOS. As shown in Fig. 2C, neither p‐eNOS Ser1177 nor p‐eNOS Thr495 was affected by PP242 treatments. Therefore, the decreased expression of Akt Ser473 exerted no effects on phosphorylated eNOS expression. Taken together, our results indicated that the phosphorylation of eNOS at Ser1177 was not related to p‐Akt Ser473 within this period.

### Inhibiting phosphorylation of Akt at Thr308 decreased NO production in HUVECs

**Fig. 3:**
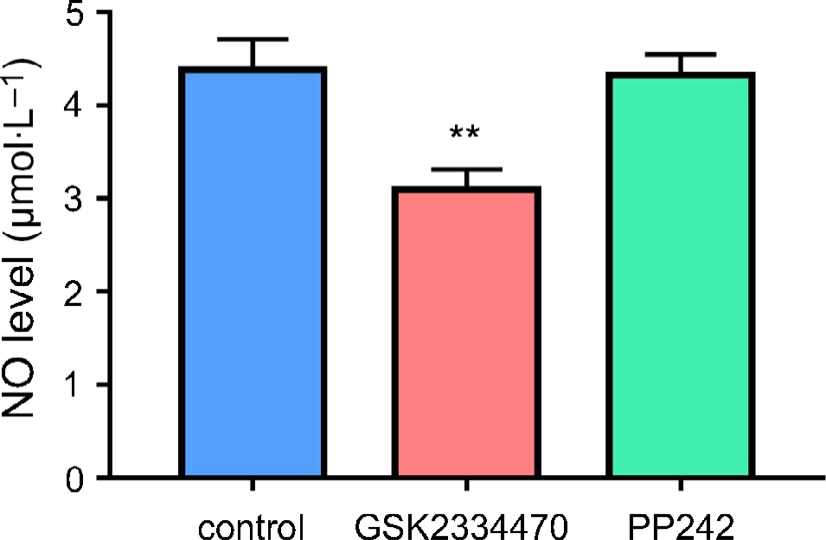
Inhibiting phosphorylation of Akt at Thr308 decreased NO production in HUVECs. HUVECs were treated with GSK2334470 (1 μm) or PP242 (100 nm) for 30 min, respectively. The NO concentration was measured. Data were expressed as mean ± SE, *n* = 4. Data were analyzed with two‐tailed unpaired Student’s t‐test. **P < 0.01 vs. Control group.

To confirm that p‐Akt Thr308 can affect the synthesis of NO, we tested the concentration of NO in the medium of cultured HUVECs. As demonstrated in Fig. 3, NO concentration was decreased to 70.7% after GSK2334470 (1 μm) treatment for 30 min, However, it remained unchanged when treated with PP242 (100 nm, 30 min).

### Knockdown of PDK1 induced the downregulation of p‐eNOS Ser1177

**Fig. 4:**
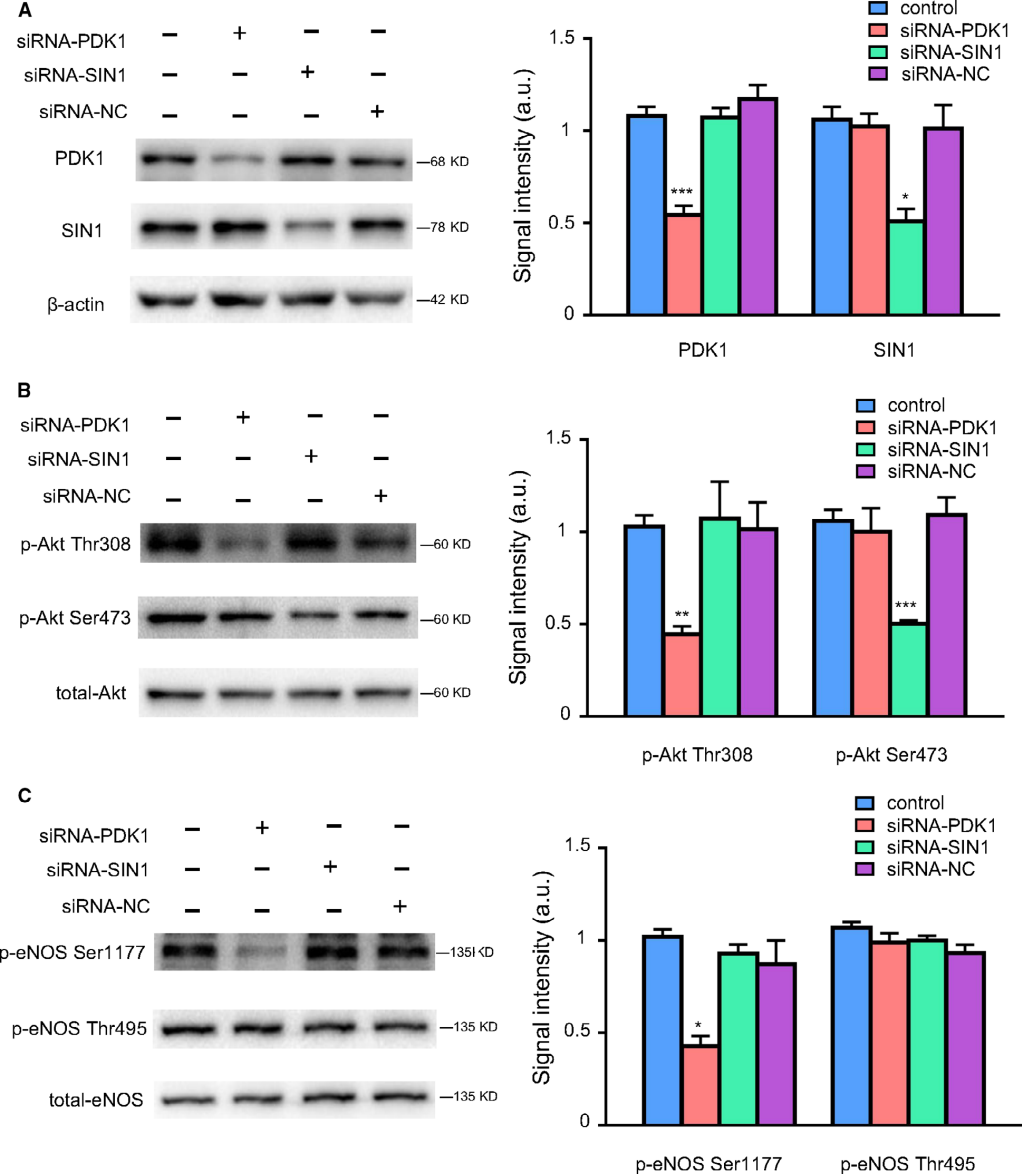
Knockdown of PDK1 induced the downregulation of p‐eNOS Ser1177. HUVECs were transfected with siRNA targeting PDK1 or SIN1 and NC scrambled siRNA (100 nm) for 48 h, the transfection efficiency as well as the total and phosphorylated levels of Akt and eNOS were detected by western blot. (A) Western blot and quantitative analysis of the protein expressions of PDK1 and SIN1 in HUVECs transfected with different siRNA oligos. (B) Western blot and quantitative analysis of the protein expressions of p‐Akt Thr308, p‐Akt Ser473, and total‐Akt in HUVECs transfected with different siRNA oligos. (C) Western blot and quantitative analysis of the protein expressions of p‐eNOS Ser1177, p‐eNOS Thr495, and total‐eNOS in HUVECs transfected with different siRNA oligos. The relative levels of p‐Akt Thr308, p‐Akt Ser473, p‐eNOS Ser1177, and p‐eNOS Thr495 were quantified as the ratios of p‐Akt Thr308/Akt, p‐Akt Ser473/Akt, p‐eNOS Ser1177/eNOS, and p‐eNOS Thr495/eNOS, respectively. Data were expressed as mean ± SE, *n* = 4. Data were analyzed with one‐way ANOVA followed by Tukey's multiple comparisons test. *P < 0.05 vs. siRNA‐NC group; **P < 0.01 vs. siRNA‐NC group; ***P < 0.001 vs. siRNA‐NC group.

SIN1 is a subunit of the upstream molecule mTORC2 leading to the phosphorylation of Akt at Ser473 [27]. To further determine the relationship between phosphorylation of Akt at different sites and eNOS activation, we used siRNA to specifically knockdown PDK1 or SIN1, respectively. As shown in Fig. 4A, the protein levels of PDK1 or SIN1 were dramatically decreased in HUVECs transfected with corresponded siRNAs. Moreover, silence of PDK1 significantly decreased the level of p‐Akt Thr308 but did not affect p‐Akt Ser473 expression, and knockdown of SIN1 markedly decreased the expression of p‐Akt Ser 473 but not p‐Akt Thr308 (Fig. 4B). Those results confirmed the specific effects of siRNA on the phosphorylation of Akt.

As shown in Fig. 4C, silencing PDK1 significantly decreased the level of p‐eNOS Ser1177, whereas SIN1 knockdown did not affect p‐eNOS Ser1177. In addition, the negative regulatory site of eNOS at Thr495 was not affected by PDK1 or SIN1 knockdown. Those data along with our previous findings strongly suggested that p‐Akt Thr308 but not p‐Akt Ser473 plays the major role in mediating the phosphorylation of eNOS Ser1177.

### Inhibition of p‐Akt Thr308 decreased p‐eNOS Ser1177 expression and NO production in mice

**Fig. 5:**
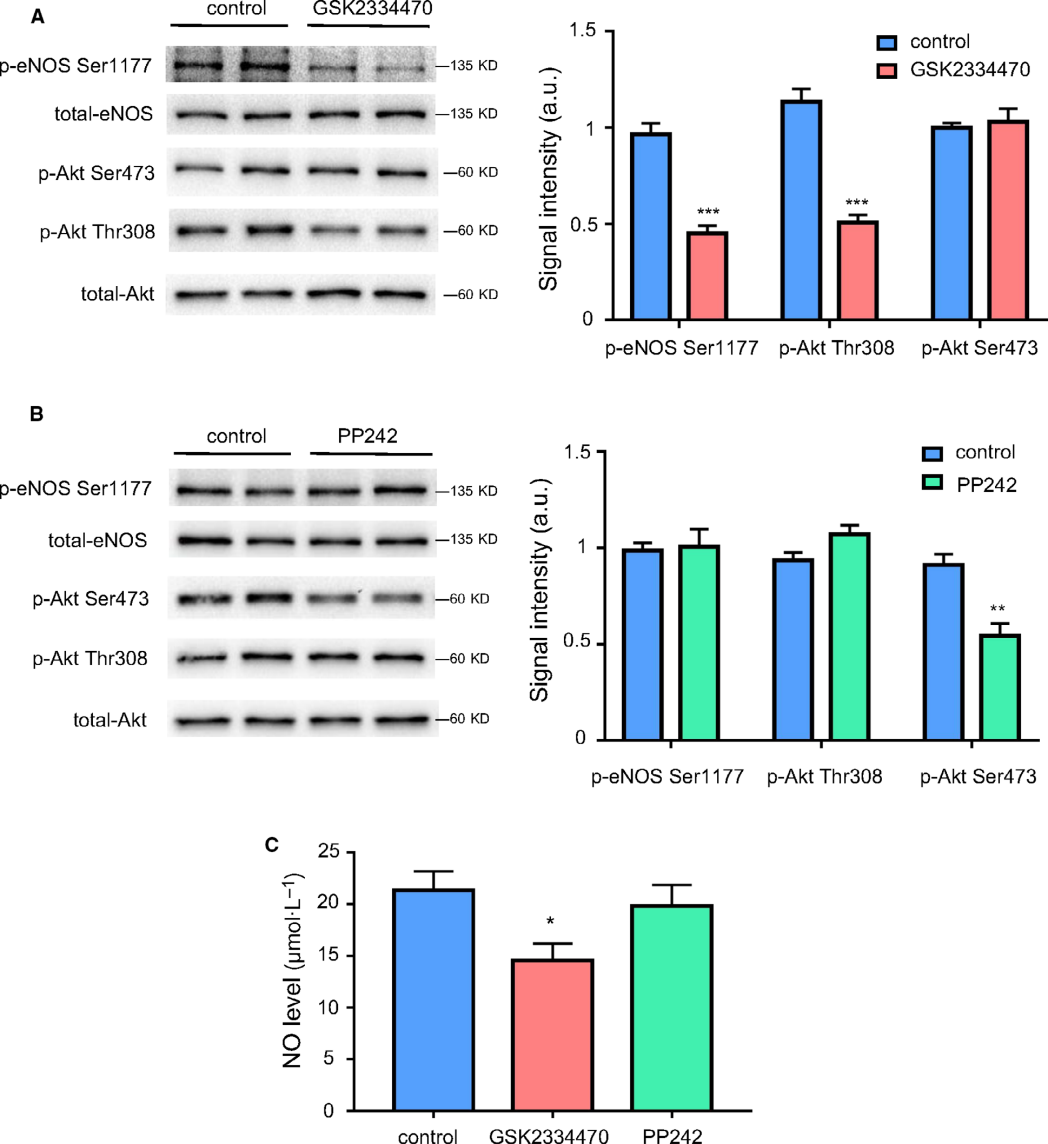
Inhibition of p‐Akt Thr308 decreased p‐eNOS Ser1177 expression and NO production in mice. Mice were administrated by a single intraperitoneal injection of GSK2334470 (40 mg·kg^−1^) or PP242 (5 mg·kg^−1^) for 6 h, respectively. The mesenteric artery of mice was harvested at the indicated time points. (A) Western blot and quantitative analysis of the protein expressions of p‐eNOS Ser1177, p‐Akt Ser473, p‐Akt Thr308, total‐eNOS, and total‐Akt in the mesenteric artery of mice treated with GSK2334470. (B) Western blot and quantitative analysis of the protein expressions of p‐eNOS Ser1177, p‐Akt Ser473, p‐Akt Thr308, total‐eNOS, and total‐Akt in the mesenteric artery of mice treated with PP242. (C) Quantitative detection of plasma NO concentration of mice treated with GSK2334470 or PP242. The relative levels of p‐Akt Thr308, p‐Akt Ser473, and p‐eNOS Ser1177 were quantified as the ratios of p‐Akt Thr308/Akt, p‐Akt Ser473/Akt, and p‐eNOS Ser1177/eNOS, respectively. Data were expressed as mean ± SE, *n* = 6. Data were analyzed with two‐tailed unpaired Student’s t‐test. *P < 0.05 vs. Control group; **P < 0.01 vs. Control group; ***P < 0.001 vs. Control group.

Above studies have demonstrated that suppression of p‐Akt Thr308 resulted in a downregulation of p‐eNOS Ser1177 *in vitro*. To further verify this effect *in vivo*, GSK2334470 or PP242 was administered to mice, respectively. As shown in Fig. 5A, mice treated with GSK2334470 (40 mg·kg^−1^, 6 h) showed significant decrease in the expressions of p‐Akt Thr308 and p‐eNOS Ser1177 in the mesenteric artery as compared with the control group; However, PP242 (5 mg·kg^−1^, 6 h), which decreased p‐Akt Ser473 expression, did not affect the phosphorylation of eNOS at Ser1177 site (Fig. 5B). As shown in Fig. 5C, accompanied the decrease in p‐eNOS Ser1177, plasma NO was obviously decreased in mice treated with GSK2334470 (40 mg·kg^−1^, 6 h), whereas it remained unchanged in mice treated with PP242 (5 mg·kg^−1^, 6 h). Taken together, these data suggested that the physiological activation and p‐eNOS Ser 1177 was mainly regulated by Akt Thr308, not Ser 473.

### p‐Akt Thr308 regulated p‐eNOS Ser1177 expression in HUVECs exposed to different temperature

**Fig. 6:**
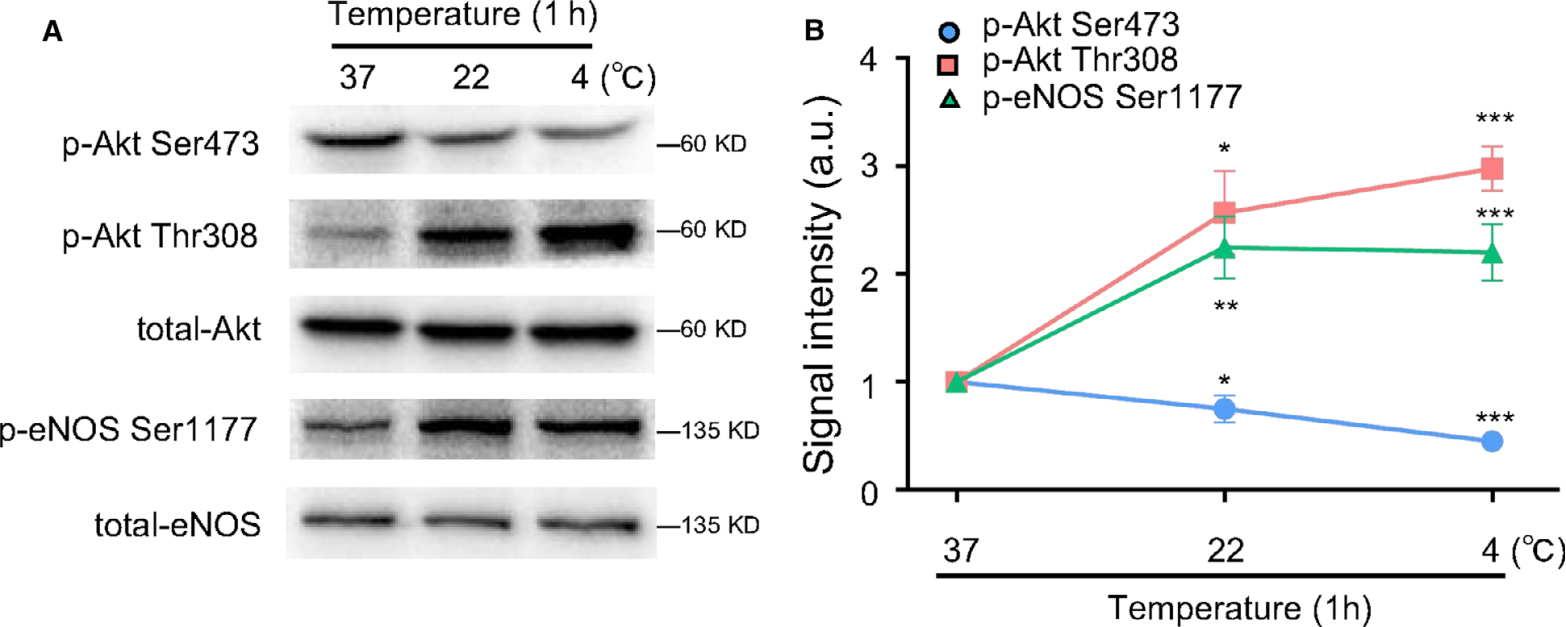
p‐Akt Thr308 regulated p‐eNOS Ser1177 expression in HUVECs exposed to different temperature. HUVECs were exposed to different temperatures (37, 22, and 4 °C) for 1 h; then the total and phosphorylated levels of Akt and eNOS were detected by western blot. (A) Western blot of the protein expressions of p‐Akt Ser473, p‐Akt Thr308, total‐Akt, p‐eNOS Ser1177, and total‐eNOS in HUVECs. (B) Quantitative analysis of the protein expressions of p‐Akt Ser473, p‐Akt Thr308, total‐Akt, p‐eNOS Ser1177, and total‐eNOS in HUVECs. The relative levels of p‐Akt Ser473, p‐Akt Thr308, p‐eNOS Ser1177 were quantified as the ratios of p‐Akt Ser473/Akt, p‐Akt Thr308/Akt, and p‐eNOS Ser1177/eNOS, respectively. Data were expressed as mean ± SE, *n* = 3. Data were analyzed with one‐way ANOVA followed by Tukey's multiple comparisons test. *P < 0.05 vs. 37 °C; **P < 0.01 vs. 37 °C; ***P < 0.001 vs. 37 °C.

Evidence has verified that room temperature and cold exposure (4 °C) could trigger the alteration of phosphorylated levels of Akt [28, 29]. To clarify the relation of different phosphorylated sites of Akt with p‐eNOS Ser1177, HUVECs were exposed to different temperature (37, 22, and 4 °C) for 1 h. As shown in Fig 6A, the gradual decrease in temperature led to a decrease in p‐Akt Ser473, whereas p‐Akt Thr308 and p‐eNOS Ser1177 were increased. The quantitative changes in phosphorylated Akt and eNOS proteins under different temperature stimulation are shown in Fig. 6B. Thus, these results indicated that the p‐eNOS Ser1177 expression was consistent with p‐Akt Thr308, but not p‐Akt Ser473.

## Discussion

As an endothelium‐derived relaxing factor, NO contributes to relax vascular smooth muscle cells, dilate blood vessels, and relief cardiovascular pain. Therefore, NO is closely related to various pathological processes of cardiovascular disorders such as atherosclerosis, hypertension, and coronary heart disease [30]. Activated Akt has been proved to stimulate endothelial cells migration, proliferation, and survival, and it serves as a key kinase that regulates NO production through activating the catalytic activity of eNOS [31]. Thus, activated Akt is usually identified as an indicator for the activity of eNOS.

Previous study demonstrated that phosphorylated sites of either Ser473 or Thr308 partially activated Akt, and both residues resulted in the maximal activation of Akt [32]. However, to date, the majority of studies focused on the phosphorylation of Akt Ser473 rather than Thr308 [33, 34, 35], probably due to the reason that antibody against p‐Akt Ser473 is relatively effective as compared with Thr308 [36]. However, which site better represents eNOS activation still remains controversial. Here, our data showed that changes in p‐Akt Thr308 were consistent with the expression of p‐eNOS Ser1177 both *in vitro* and *in vivo*, and the loss of p‐Akt Thr308 resulted in impaired NO production. However, p‐eNOS Ser1177 changes independently with p‐Akt Ser473. In addition, we found that p‐Akt Ser473 did not affect the expression of p‐eNOS Ser1177 in HUVECs under different temperature, further indicating that the phosphorylated site of Akt at Thr308, but not Ser473, regulated p‐eNOS Ser1177 expression under physiological conditions.

Our study showed that p‐Akt Ser473 exert rare effects on p‐eNOS Ser1177. Consistent with our observation, Estela Jacinto and David A. Guertin have proved that selectively inhibiting the phosphorylation of Ser473 weakened the downstream substrates FOXO1/2a and FOXO3, whereas it did not affect GSK3β and TSC2, which are all supposed to be regulated by Akt. Those findings suggest that phosphorylation of Ser473 may determine the substrate specificity of Akt but do not affect its activity [37, 38]. A recent study also pointed out that phosphorylation of Ser473 alone exerted limited effect on Akt activity [32]. Similarly, blocking phosphorylation of Ser473 did not affect Akt activity in human platelets [39]. These data convincingly suggest that phosphorylation of Ser473 may not be a direct trigger of Akt activation.

Nevertheless, some studies are inconsistent with our findings. Previous study showed that inhibitor of G protein‐coupled receptor kinase 2 (GRK2) enhanced the expression of p‐eNOS Ser1177 through upregulating both p‐Akt Ser473 and Thr308 in diabetic mice [40]. Romic's *et al*. found that a diet rich in fructose resulted in decline of p‐Akt Thr308 and p‐Akt Ser473 as well as p‐eNOS Ser1177 [41]. These studies indicated that trends in p‐Akt Thr308 and Ser473 were broadly consistent, and they both regulated p‐eNOS Ser1177 expression. Those inconsistency may be related to the different time effect. Our study paid close attention to detect p‐eNOS Ser1177 expression in a short term, which possibly helped to reveal the potential intrinsic links between phosphorylation of Akt and eNOS activation under physiological condition.

As a negative regulatory site that inhibits eNOS activation, p‐eNOS Thr495 was not modulated by Akt, but mainly regulated by AMP‐dependent protein kinase (AMPK) and protein kinase C (PKC) signal transduction pathways [42]. In contrast to Thr495, Ser1177 can be catalyzed by a variety of kinases, including Akt, protein kinase A (PKA), AMPK, protein kinase G (PKG), and calmodulin kinase II (CaM kinase II) [16, 43, 44, 45, 46]. Among them, Akt is a key factor in the regulation of p‐eNOS Ser1177. Considering that eNOS activation is mainly dependent on the phosphorylation of Ser1177, we also determined the effects of p‐Akt Thr308 on NO production in physiology. Our results showed that phosphorylation of Akt at Thr308 equally plays a crucial role in NO production both *in vitro* and *in vivo*, revealing an important role of p‐Akt Thr 308 in maintaining vascular tone. Although only HUVECs were used in the cell experiments of the present study, to our knowledge, the other types of endothelial cells derived from different vascular tissues (veins or arteries) or different calibers (aorta or arteriole) exert similar Akt phosphorylation pattern [47, 48, 49].

In addition to regulating NO production, p‐Akt exerts multiple effects on endothelial cells function, such as angiogenesis and vascular remodeling [50, 51, 52]. As an important signaling pathway, PI3K/Akt/eNOS axis mediates PGI2 release and COX‐2 expression induced by HDL [53]. Moreover, Lin’s study found that visfatin promoted monocyte‐endothelial cell adhesion by increasing ICAM‐1 and VCAM‐1 expression via p38/PI3K/Akt signaling [54]. Therefore, Akt signaling plays a complex, critical, and multifunctional role in endothelial cells functions, which need to be further explored in the further study.

## Conclusion

In summary, our study indicates that phosphorylation of Akt at Thr308 site specifically regulates p‐eNOS Ser1177 expression, and Thr308 is a powerful phosphorylation site which determines Akt activity and judges the trend of variations in Akt downstream targets.

## Conflict of interest

Xiao‐xue LIANG, Rui‐yu WANG, Yong‐zheng GUO, Zhe CHENG, Ding‐yi LV, Ming‐hao LUO, An HE, Su‐xin LUO and Yong XIA declare that they have no conflict of interest.

## Author contributions

YX and SXL conceived and designed the project. XXL, ZC, DYL, MHL, and AH performed the experiments. XXL and YX analyzed and interpreted the data. XXL, RYW, and YZG wrote the article. All authors have read and approved the final manuscript.

## Data Availability

The data that support the findings of this study are available from the corresponding author [yong.xia@osumc.edu] upon reasonable request.
